# Knowledge and perceptions of risk for cardiovascular disease: Findings of a qualitative investigation from a low-income peri-urban community in the Western Cape, South Africa

**DOI:** 10.4102/phcfm.v7i1.891

**Published:** 2015-10-22

**Authors:** Sam Surka, Krisela Steyn, Katherine Everett-Murphy, Thomas A. Gaziano, Naomi Levitt

**Affiliations:** 1 Chronic Disease Initiative for Africa, Cape Town, South Africa; 2 Brigham and Women's Hospital, Harvard University, United States; 3 Department of Medicine, University of Cape Town, South Africa

## Abstract

**Background:**

South Africa currently faces an increasing burden of cardiovascular disease. Although referred to clinics after community screening initiatives, few individuals who are identified to be at high risk for developing cardiovascular disease attend. Low health literacy and risk perception have been identified as possible causes. We investigated the knowledge and perceptions about risk for cardiovascular disease in a community.

**Method:**

We conducted a series of focus group discussions with individuals from a low-income peri-urban community in the Western Cape, South Africa. Different methods of presenting risk were explored. The data were organised into themes and analysed to find associations between themes to provide explanations for our findings.

**Results:**

Respondents’ knowledge of cardiovascular disease and its risk factors varied, but most were familiar with the terms used to describe cardiovascular disease. In contrast, understanding of the concept of risk was poor. Risk was perceived as a binary concept and evaluation of different narrative and visual methods of presenting risk was not possible.

**Conclusion:**

Understanding cardiovascular disease and its risk factors requires a different set of skills from that needed to understand uncertainty and risk. The former requires knowledge of facts, whereas understanding of risk requires numerical and computational skills. Without a better understanding of risk, risk assessments for cardiovascular disease may fail in this community.

## Abstract (fr)

**Connaissance et perceptions des risques de maladies cardiovasculaires: résultats d’une étude qualitative dans une communauté périurbaine à faibles revenus du Western Cape, en Afrique du Sud.**

**Contexte:**

A l’heure actuelle, l’Afrique du Sud se trouve confrontée à une augmentation des maladies cardiovasculaires. Bien que les patients soient envoyés dans des cliniques suite à des initiatives communautaires de dépistage, peu de ceux qui sont identifiés à haut risque de contracter des maladies cardiovasculaires se présentent. La raison en est probablement les faibles connaissances en santé et en perceptions des risques. Nous avons examiné la connaissance et la perception des risques de maladies cardiovasculaires dans une communauté.

**Méthode:**

Nous avons mené une série de discussions de groupes témoins avec des individus d’une communauté périurbaine à faibles revenus du Western Cape, en Afrique du Sud. Nous avons examiné différentes méthodes de présentation de risques. Les données ont été classées par thèmes et analysées pour trouver des rapports entre les thèmes afin d’expliquer nos résultats.

**Résultats:**

La connaissance des personnes interrogées sur les maladies cardiovasculaires et leurs facteurs de risques différaient, mais la plupart d’entre elles connaissaient la terminologie utilisée pour décrire les maladies cardiovasculaires. Par contre, elles comprenaient mal le concept de risque. Le risque était perçu comme une idée binaire et l’évaluation des différentes méthodes narratives et visuelle de présentation des risques n’était pas possible.

**Conclusion:**

Pour comprendre les maladies cardiovasculaires et leurs risques il faut avoir un ensemble de compétences différentes de celles requises pour comprendre l’incertitude et les risques. Le premier demande une connaissance des faits, alors que la compréhension des risques exige des compétences numériques et informatiques. Sans une meilleure connaissance des risques, l’évaluation des maladies cardiovasculaires peut échouer dans cette communauté.

## Background

South Africa currently faces an increasing burden of cardiovascular disease (CVD).^1^ Primary prevention of CVD, based on the early identification and treatment of high-risk individuals, is a well-established strategy aimed at reducing the rising burden of these conditions globally.^2,3,4^ Identification of high-risk individuals involves screening the general population for their total CVD risk, typically using a risk assessment tool.^5,6,7,8^ A non-laboratory-based risk assessment tool, in which a blood lipogram is replaced by using a body mass index value in determining a risk score, obviates the need for laboratory infrastructure and blood tests and, as a result, can lower the cost of these screening procedures.^6^ This risk assessment tool has been validated in South African populations and has been found to be effective in identifying individuals at high risk of developing CVD.^6,9^ In addition, community-based screening has been investigated as a means to further scale up this component of the primary prevention intervention process. In this regard, it has been found that community health workers (CHWs) are able to accurately conduct risk assessments in community settings after undergoing a training programme.^10,11^

However, when at-risk individuals are referred to the primary healthcare facility by a CHW for formal assessment and further management, the attendance level is low.^12^ Numerous reasons for low attendance have been identified, including low health literacy and poor understanding of the concept of having a high risk for CVD. Although providing an individual with their CVD risk score has been found to be a useful way to motivate healthy behaviour,^13,14^ it can also be challenging for patients with low health and numerical literacy.^15,16,17^ Diverse demographic, socio-psychological and cultural variables also influence an individual's perception of risk and can affect health-related behaviour choices.^18^

Health literacy refers to an individual's cognitive and social skills, which determine their ability to access, understand and use information in ways that promote and maintain good health.^19^ Low levels of health literacy are associated with poorer health outcomes, less use of health services and less involvement in self-management of chronic conditions.^20^ In addition, according to behaviour theories such as the health belief model, the perceived severity of and susceptibility to a disease, as well as perceived benefits and barriers to changing health behaviour, can influence an individual's health-seeking behaviour.^21^ Improving the primary healthcare attendance of individuals identified as being at high risk may require designing interventions that take into consideration the baseline health and numerical literacy of the target population. It will also require addressing the determinants of health-seeking behaviour as identified in known behaviour change theories.

In the context of health, risk can be defined as a measure of the chance that damage to health or loss of life will occur as a result of a particular hazard. In the context of CVD, this refers to the likelihood of events such as heart attacks or strokes, whether fatal or non-fatal. Much research in developed countries has focused on evaluating the type of visual presentation of risk (e.g. bar graphs, risk tables, heart age, etc.) that is most effective in communicating risk.^15,22,23,24,25^ Understanding and being able to interpret risk is an important component of how one determines health-related behaviour and therefore ensuring that patients understand risk should be an essential component of any intervention that aims to screen individuals for CVD risk. To our knowledge, no previous study has investigated how CVD risk is understood in a low-income peri-urban setting in South Africa, nor which methods of risk presentation are most effective. This qualitative investigation aimed to deepen our understanding of knowledge and perceptions about CVD and its risk factors in this community and to explore how the concept of risk can best be presented.

## Methods

### Study design

A qualitative study design was used to explore the perceptions of community members in greater depth. Focus group discussions (FGDs) were chosen to encourage interaction between the respondents in order to spark further discussion and draw out latent perceptions and ideas around the topic of CVD and risk.

### Setting and respondents

The study was conducted between May 2014 and July 2014 in the Nyanga township outside Cape Town in the Western Cape, South Africa. Nyanga has close to 16 000 households and a total population of about 58 000. The population is predominantly black African (99%), of which only 31% have completed secondary schooling (Grade 12). The unemployment rate is high (45%) and the average monthly income is about R3200 (approximately $320 at the time of the study) (National Census data, 2011). In recruiting community members for this study, we returned to the population in which we previously studied community-based risk assessment and in which poor CVD risk perception was identified as a reason for not attending community health centres after referral.^12^ Purposive sampling was conducted by asking CHWs to recruit male and female community members (≥ 25 years) of the Nyanga township and who had no previous experience in being assessed for CVD risk. Individuals with a past history of CVD were not excluded and CHWs were allowed to recruit their existing clients. Participation was voluntary and each respondent was informed about the study objectives a week before the discussions were scheduled to take place. Prior to the FGDs, a total CVD risk score was calculated for each respondent using the non-laboratory CVD risk assessment tool and communicated to them by the CHWs. The method used to calculate these risk scores has been described previously.^11^

### Data collection

We conducted three FGDs in groups consisting of between 8 and 10 respondents at a time. An experienced facilitator, fluent in the first language of the respondents but who was not from the community, conducted the discussions. Key questions were developed and respondents were asked about their knowledge, perception and understanding of various terms used to describe CVD (including heart attack, stroke, risk, risk factors and cardiovascular disease). They were also asked about causes and risk factors of CVD, modifiable risk factors, prevention, risk factors, risk factor modification, risk scores, and their preference for risk score presentation. A number of different narrative and visual methods of presenting the concept of risk were explained to the respondents. These methods included presenting risk scores, a risk chart, a pictograph and explaining the concepts of relative risk and heart age (see Table 1 for a detailed description). Respondents were asked to rate each method using a Likert scale, where 1 denoted ‘easy to understand’ and 5 denoted ‘difficult to understand’. Respondents were also encouraged to raise other topics or issues related to cardiovascular health. At key intervals in the discussion, information was summarised by the facilitator and repeated to the respondents to ensure that it accurately reflected their intended message. The FGDs were conducted in isiXhosa and the discussions were recorded digitally. Each discussion lasted approximately 60–90 minutes. An experienced translator transcribed all the data verbatim into English.

**TABLE 1:** The methods used to explain the concept of risk of developing cardiovascular disease.

Method of risk presentation	Explanation	Visual aid description
Five-year risk score	Your risk score is 30%. This means you have a 30% chance of ha heart attack or stroke in the next five years.	None.
Risk chart	Your risk score is 30%, which means you have moderate to high risk of having a heart attack or stroke in the next five years.	A graded, colour-coded risk chart (with low risk represented in blue, moderate risk in yellow and high risk in red) is used to show where an individual's risk lies.
Pictograph (100-face diagram)	Your risk score is 30%. In other words, in a crowd of 100 people with the same risk factors as you, 30 are likely to have a heart attack or stroke within the next five years.	A diagram depicting a hundred faces (70 smiling and coloured in yellow; 30 not smiling and coloured in blue) is used to illustrate this concept.
Relative risk	Your risk score is 30%. The score of a typical person of the same age, gender and ethnicity is 6%. Your relative risk is therefore 5. This means that you are five times more likely to have a heart attack or stroke in the next five years than a typical person of the same age, gender and ethnicity as yourself.	None.
Heart age	Although your actual age is 35 years, your ‘heart age’ is 40 years. This means that you have the same risk (of having a heart attack or stroke) as someone who is 40 years old. In other words, your heart is less healthy than it should be at your age.	None.

## Data analysis

The data were explored and analysed following the qualitative analysis framework described by Pope et al.^26^ The transcripts were first reviewed in their entirety several times by the first author and organised into themes. All key issues and themes were examined and referenced. The transcripts were annotated and the data were indexed according to the identified themes to obtain an overall picture. The data were analysed to find associations between themes to provide explanations for the findings. Selected quotations from respondents have been included in this report to illustrate the emergent themes. These are reported without identifiers to preserve the anonymity of respondents. A post hoc analysis of the FGD findings was performed within the framework of the health belief model.

## Ethical considerations

Ethical approval for this study was obtained from the Human Research Ethics committee (University of Cape Town). Written information about the study was provided and informed consent was obtained from respondents for participation and having their CVD risk scores calculated. Individuals identified as being at high risk of CVD were provided with a referral letter for follow-up at the nearest community health centre.

## Results

We enrolled 30 respondents to participate in 3 FGDs; however, 2 respondents did not attend their allocated sessions. There were 4 male and 24 female respondents. Although the exact reasons for low recruitment and participation of male respondents in this study are unknown, it is in keeping with the skewed gender participation in previous CHW-led initiatives conducted in this community.^10,12^

The average age of the respondents was 53 years (± 16 years). The average level of education was nine years of schooling (grade 9).

Of the total sample, 14 respondents reported a history of hypertension, 3 reported ischaemic heart disease and 2 had a history of cerebrovascular disease. No respondents reported diabetes. A non-laboratory-based risk assessment tool was used to assess each individual's risk for having a cardiovascular event (either fatal or non-fatal) within the next five years. The following levels of risk were identified: 14 respondents were found to have low risk, 4 presented with low to moderate risk, 8 presented with moderate risk, one had moderate to high risk and one had high risk. The CVD risk profile of respondents in this study was higher than found in previous studies conducted in this population.^27,28,29^ This result can likely be attributed to the purposive recruitment of existing CHW clients and the non-exclusion of individuals with a medical history of CVD.

The results of the FGDs are presented according to the three themes and respective categories shown in Table 2.

**TABLE 2:** Main themes and categories discussed in the focus group discussions.

Themes	Categories
Knowledge of CVD and its prevention	Cardiovascular disease. Myocardial infarction. Cerebrovascular accidents. Hypertension. Preventative measures.
Perception of risk	Concept of risk. Risk factors of CVD. Risk scores. Risk presentation.
Coping with the disease	Attitude towards making lifestyle changes. Personal, cultural and religious beliefs.

CVD, cardiovascular disease.

### Knowledge of cardiovascular disease and its prevention

#### Cardiovascular disease

Across all FGDs respondents were hesitant and unsure when asked what they thought this term meant and were unable to provide a definition. Some respondents described CVD as conditions affecting the heart or identified specific conditions such as hypertension and diabetes. Many had heard of conditions associated with CVD but felt uninformed owing to poor communication from health care providers:

‘We do understand this question, but we do not know these diseases because the doctor does not explain. He just tells you that you have diabetes or hypertension, but does not explain where do you get them and what are you supposed to do. They [*are*] supposed to tell us but they do not.’ (Respondent 1, FGD 2)

#### Myocardial infarction

All respondents were familiar with the term ‘heart attack’, with some describing it in more general terms as ‘a heart problem’, whereas others provided a more detailed explanation:

‘… when blood is not circulating properly; when it [*a blood vessel*] is blocked that it can cause [*a*] heart attack, maybe when there is too much fats in the veins and blood is not flowing properly.’ (Respondent 2, FGD 1)

‘His heart is not functioning normally … it either has irregular beats or it can stop at any time.’ (Respondent 1, FGD 1)

#### Cerebrovascular accidents

Respondents were also familiar with the term ‘stroke’. Many of the respondents personally knew people who had experienced a stroke and understood the debilitating consequences. When asked what they thought the possible causes are, some mentioned the link between having a stroke and having high blood pressure; others thought there was a progression from hypertension to asthma to having a stroke. Some respondents thought that ‘thinking too much can cause strokes’ as well as going into ‘shock’ after hearing tragic news:

‘Thinking too much can cause [*a*] stroke because you think too much and get lost in your thoughts to the point that you can’t even hear a person talking to you.’ (Respondent 3, FGD 2)

Some respondents were sceptical of the diagnosis of stroke in people who were otherwise healthy and without any illnesses:

‘The doctors diagnosed her with [*a*] stroke but that sounded creepy to me because she never complained about any pains in her body and she was not one of the people who are taking medication, and she was never diagnosed with high blood pressure. She eventually died …’ (Respondent 6, FGD 2)

#### Hypertension

This was also a condition many respondents were familiar with. However, none were able to provide a clear definition or explanation of what hypertension is – even those who had been diagnosed as hypertensive. One respondent explained that despite being on anti-hypertensive treatment, she did not have any real insight into her treatment and was doing only what was advised by her doctor:

‘They say I have high blood pressure and I am taking these tablets, which I do not even know what they are for. I am just following orders. I do not have a clue about all of this.’ (Respondent 7, FGD 2)

Another respondent associated her diagnosis of hypertension with stress and expressed frustration in not understanding the causative link:

‘I was diagnosed with high blood [*pressure*] in 2002. It started with stress. I used to collapse and people would pour water to wake me up … The doctors said my stress levels were very high. I would also have a severe headache. I have a problem of stress and the doctors recommended that I should have [*watch*] TV or play cards, just something to take my mind off things. Sometimes it feels like I am going crazy. We have these diseases and we keep asking ourselves why are we suffering from these diseases.’ (Respondent 5, FGD 1)

Respondents noted that poor adherence to anti-hypertensive treatment could also put an individual at risk of a stroke:

‘You don’t take treatment for high blood pressure and you end up having [*a*] stroke. Then that means it can put you at risk, or when you were not complying with your treatment. Maybe you’re mixing or confusing treatment time. Then the doctor can tell you that you are at risk because you were not taking your treatment regularly.’ (Respondent 4, FGD 2)

Other causes of CVD identified by respondents included depression and ‘talking too much’, which refer to situations that cause emotional distress and stress:

‘Well, when I say “talking too much” I can mention grandchildren because we have grandchildren. Our grandchildren do not listen to us; they do this today and do something else tomorrow. So when they do these things you always talk, trying to discipline the child, and the child responds negatively and that hurts you. Your heart does not relax.’ (Respondent 4, FGD 2)

#### Preventative measures

Only modification of diet, smoking cessation and reducing alcohol consumption were identified as preventative actions that could be taken to prevent CVD. No mention was made of hereditary factors, high cholesterol, sedentary lifestyle or obesity:

‘I understand that if you have [*a*] heart problem and high blood [*pressure*], you must not feed your body with everything. You can pretend but your blood can detect that …’ (Respondent 3, FGD 1)

### Perceptions of risk

#### Concept of risk

The majority of respondents were unable to provide a definition or explanation of what they understood by the concept of risk. Only two respondents across the three FGDs were able to give a correct example of where the word ‘risk’ was used as pertaining to CVD; however, they were unable to explain what they thought it meant. In both cases, respondents explained how the term was used to convey that because of poor adherence to medication they were ‘at risk’ of having a stroke.

Many respondents were unclear about why some people presenting with risk factors become ill, whereas others do not:

‘They say you [*get*] [*an*] ulcer because of alcohol. I do not know about me, because I am not drinking, and I never touched a glass of alcohol but I do have these diseases [*ulcers*].’ (Respondent 4, FGD 1)

#### Risk factors of cardiovascular disease

Respondents understood that there were certain behaviour-related factors that contribute to developing heart disease and stroke. Tobacco smoking, excessive alcohol consumption and not being compliant with regard to medication use for hypertension were mentioned as contributing factors. Unhealthy diets were identified as a major contributing factor and respondents specifically mentioned fatty meat and salty food as being unhealthy:

‘… when you eat red meat too much [*often*], because the fat in red meat will be deposited in the arteries and block the blood flow …’ (Respondent 3, FGD 1)

Many respondents also mentioned that certain methods of preparation make meat less unhealthy (trimming the fat and grilling were given as examples). Some specifically linked unhealthy diets to conditions such as hypertension and diabetes and referred to the increasing prevalence of these conditions compared to when more traditional diets, with smaller portions and prepared with less oil, were consumed:

‘We are no longer eating traditional food … people from that era were not developing diabetes and hypertension and these diseases were not that popular … people were consuming fresh food from their gardens and they were not interested in fatty foods. They were eating indigenous food only. But here in town everything is cooked by [*in*] oil, and nobody is educating us about the portion sizes. We think we are eating health[*il*]y, but we are killing ourselves.’ (Respondent 3, FGD 2)

Respondents also associated unhealthy diets with their circumstances and with poverty in general. They noted that despite being aware of having to eat more fresh fruit and vegetables and less fatty foods, they were not always able to do this owing to the cost associated with procuring leaner meats and fresh produce:

‘Our living situation forces us to eat whatever is available. Sometimes you know you do not want to eat this, but you are forced to eat it. We do not have vegetables and we do not have land to do small gardens.’ (Respondent 6, FGD 1)

All respondents were aware that tobacco smoking and excessive alcohol consumption are risk factors for heart disease:

‘… there is alcohol that kills people. Some drink excessively and they end up developing heart problems …’ (Respondent 4, FGD 2)

The type of alcohol being consumed in peri-urban settings was also discussed as being more harmful than that consumed by individuals of other socio-cultural backgrounds:

‘If I am drinking, I must drink like white people, because when they are drinking, they dilute their alcohol [*spirits*], unlike us; we just drink it raw as it is [*without the addition of a mixer*].’ (Respondent 4, FGD 1)

In addition, some respondents identified cultural and religious beliefs, such as ‘dark spirits’ or a deity, as the cause of CVD. Most respondents, particularly in focus group 1, agreed with these attributions.

#### Risk scores

When asked whether they considered themselves to be at risk of developing CVD, based on their calculated risk score, respondents were unable to associate a high risk score with a higher likelihood of developing CVD. In general, respondents interpreted the risk score to have a binary outcome: they were either going to develop CVD (if they had a high score) or not (if they had a low score). Only one respondent, who was found to be at high risk, explained that because she had a high risk score, she had ‘more chances of contracting these diseases’. Most respondents did not know how to interpret the risk scores, stating that ‘anything is possible’. This suggests that irrespective of the risk scores calculated for each individual, there was no change in the perceived susceptibility of having a cardiovascular event.

#### Risk presentation

We were unable to collect any useful information with regard to comparing different methods of presenting risk scores during the FGDs. Respondents did not understand the word ‘risk’ and subsequently did not understand any of the methods of presenting and explaining risk. During the FGDs, we directly observed that, despite the translator explaining each method a number times, respondents remained unsure and were unable to score the different methods.

### Coping with the disease

#### Attitude towards making lifestyle changes

Respondents felt helpless about making lifestyle changes. This was largely attributed to dire living conditions and poverty:

‘… and sometimes you do not have a cent to buy vegetables from the street vendors. I went to see a doctor and the doctor said: “Your blood pressure is high; therefore you need to eat fruit and vegetables.” I asked him: “Doctor, where will I get these nutritious foods?” I have nothing. I am surviving on [*a*] child's grant of R300 [*a month*].’ (Respondent 6, FGD 1)

‘Poverty can cause these diseases. You become helpless. You do not know where to start.’ (Respondent 6, FGD 2)

### Personal, cultural and religious beliefs

Respondents also referred to a number of personal, cultural and religious beliefs that influenced how risk was perceived. For example, respondents mentioned that once diagnosed with CVD, progression was beyond their control:

‘It is only God who can help us … ’ (Respondent 2, FGD 1)

## Discussion

Understanding diseases and their predisposing factors requires a different set of skills from that needed for understanding uncertainty and risk. The former requires knowledge of facts and that certain behaviours or biological processes can lead to specific outcomes, such as heart attacks. However, understanding risk requires abstract thinking that involves numerical literacy and computation. For example, risk scores are often numeric expressions of uncertainty about a future event. Being able to understand, interpret and work with numbers leads to improved risk comparisons and estimates,^30^ whereas a lower level of numeracy skill is associated with an overestimation of risk as people tend to respond more to non-numerical aspects of the interaction, such as mood or feedback from others.^31,32^

In this study, respondents’ knowledge of CVD and its risk factors varied, but most were familiar with the terminology used to describe CVD. For example, respondents were aware of some lifestyle factors that cause CVD, such as high-fat diets and excessive alcohol consumption, which suggests that there is some baseline knowledge of this topic amongst community members. Similarly, respondents were aware of a number of cardiovascular conditions, such as hypertension and stroke. However, although they were familiar with the terms, they had limited insight into the conditions. The limited insight was underscored by contrasts in the self-reported history of CVD and the calculated level of risk, which suggests low health literacy amongst respondents. An exception was respondents’ awareness of the impact of living in low-income peri-urban conditions on the risk of developing CVD. This insight stemmed from the comparison with people living in more rural settings, where fewer people were perceived to develop CVD, and is in keeping with the evidence on the rising incidence of CVD associated with urbanisation.^33^

In contrast to having knowledge of CVD and its risk factors, respondents were altogether unfamiliar with the concept of risk. Risk was perceived as a binary concept, in that a cardiovascular event was either going to happen or not, depending on high or low risk, respectively. This finding is in keeping with those of other studies that explored how CVD risk is understood.^18,34,35^ One of the objectives of this study was to explore different methods of conveying risk. Although the difficulty in understanding CVD risk tables and multivariable risk factors has previously been documented in the literature,^17^ we underestimated the extent to which it would restrict our investigation. Consequently, we were unable to assess the effectiveness of different methods owing to respondents’ lack of primary understanding of risk. Despite evidence to support the use of certain strategies to improve risk communication in general (e.g. presenting the chance of an event, presenting changes in numeric outcomes and using visual formats),^31^ more research is needed with regard to developing methods for communicating risk to people with little numerical literacy.

Being able to understand risk is only one of the factors that influence how people understand and use information in their decision-making. Several theories regarding decision-making have been put forward, particularly related to risk.^18^ The health belief model, developed to explain why people fail to accept screening tests for early detection of asymptomatic disease,^36^ suggests that people are unlikely to take appropriate preventative action until they perceive themselves to be susceptible to developing a disease associated with serious consequences. We analysed the findings from the FGDs according to the framework of the health belief model to evaluate CVD risk assessment as a possible driver of behaviour change (Table 3 and Table 4). Although CVD risk assessment supports a number of the constructs of the proposed model (e.g. perceived susceptibility, perceived severity, perceived benefits of taking action and a cue to action), it still requires health and numerical literacy.^37^

**TABLE 3:** Key descriptors of the health belief model as described by Rosenstock (1974).^21^

Key descriptors	Explanation
Perceived susceptibility	The perception of the likelihood of experiencing a given disease or condition.
Perceived severity	The perception of the seriousness of the effects a given disease or condition would have on one's state of affairs, including the emotional and financial effects.
Perceived benefits of taking action	The perception of the benefits to be gained by taking an action.
Barriers to taking action	Barriers relate to the inhibitory characteristics of a treatment or preventative measure that prevent action, including, for example, the inconvenience or expense.
Cue to action	The cue to action acts as a trigger for the desired action to be taken.

**TABLE 4:** Analysis of the findings from focus group discussions and evaluation of the risk for cardiovascular disease within the framework of the health belief model.

Key descriptor	Findings from FGDs	Responses	Evaluation of CVD risk assessment as an intervention to drive behavioural change
Perceived susceptibility	A limited understanding exists of the causal relationship between CVD and its risk factors.	‘… it [*stroke*] starts with high blood pressure and then asthma …’	The calculation of a CVD risk score provides information that directly informs perceived susceptibility (i.e. a risk score directly communicates the likelihood of an individual having a cardiovascular event within a given period).
	The absence of external symptoms, as in the case of hypertension, negatively affects perceptions of susceptibility.	‘… my blood pressure is high ever since last month … I am at peace with it. Some things are part and parcel of our lives and you just do not know what to do and end up destroying your heart.’	
	Perceived susceptibility remained unchanged by calculation of a cardiovascular disease risk score.	‘Anything is possible.’	
Perceived severity	The physical severity of consequences of CVD was perceived adequately.	‘Back home in [*the*] Eastern Cape there is a woman who is suffering from stroke. She cannot even do a thing for herself …’ ‘…she was attacked by stroke when we were at church … She eventually died.’	The calculation of a CVD risk score provides information that indirectly informs perceived severity (i.e. the risk score communicates the likelihood of having a stroke or heart attack in the given period).
	Perceptions on the psychosocial, emotional and financial effects were not discussed.		
Perceived benefits of taking action	Awareness of the benefits of taking action was limited. Even respondents who were taking action (e.g. adhering to treatment) did not express perception of any benefits gained.	‘It is also the beliefs. We believe that these tablets can cause cancer so you decide to stop taking them. And when you look at the amount [*sic*] of these pills you believe that it is true that you can develop cancer.’	The benefit of taking action (by modifying risk factors) is the reduction of risk and potential prevention of a heart attack or stroke. This is not currently quantified as part of the risk assessment (e.g. explicitly stating that smoking cessation for example, will reduce risk by 10%).
	Cultural, religious and other beliefs further detracted from the perceived benefits of taking action.		
Barriers to taking action	Poverty was identified as a barrier to making lifestyle changes.	‘… our living situation forces us to eat whatever is available …’	CVD risk assessment does not address barriers to taking action.
Cue to action	None elicited.		Once identified as being at high risk, a referral to the local clinic can be considered a cue to action.

CVD, cardiovascular disease; FGD, focus group discussion.

The ability to understand risk may be further complicated by an element of fatalism. Respondents expressed a sense of helplessness (‘… poverty can cause these diseases; you become helpless …’; ‘… it is only God who can help us’), which suggests that they perceive an external locus of control and that they cannot control events affecting their lives.^38^

There are a number of limitations in this study. A selection bias was introduced by CHWs recruiting their existing clients, including those with a previous history of CVD. As the majority of respondents were female, a skewed distribution of gender resulted. The data were analysed by a single researcher, which may have introduced bias in interpretation. Language and cultural differences between the researcher and the respondents may also have affected understanding and interpretation of the data. In addition, respondents’ numeracy skills were not determined and inferences in the discussion were based on the overall level of completed secondary schooling.

## Conclusion

This study contributes to our understanding of the knowledge of and perceptions about CVD and its risk factors in a low-income peri-urban community in South Africa. Respondents were more knowledgeable about CVD in general than about the concept of risk. Educational initiatives focused on improving knowledge of risk and the causal relationship between predisposing factors and CVD are needed. These need to consider local cultural and religious beliefs. In addition, methods to present risk without numeracy skills being necessary for interpretation also need to be developed and tested in this community. Without a better understanding of risk, CVD risk assessments may fail in this community.
